# Epidemiological Survey of DNA Viruses in Non-Native Pond Sliders (*Trachemys scripta*) in Northeastern Italy

**DOI:** 10.3390/v18060676

**Published:** 2026-06-17

**Authors:** Claudia Maria Tucciarone, Giovanni Franzo, Daniela Pasotto, Riccardo Baston, Luca Spadotto, Cinzia Centelleghe, Erica Marchiori

**Affiliations:** 1 Department of Animal Medicine, Production and Health (MAPS), University of Padua, Viale dell’Università 16, 35020 Legnaro, Italy; claudiamaria.tucciarone@unipd.it (C.M.T.); giovanni.franzo@unipd.it (G.F.); erica.marchiori@unipd.it (E.M.); 2 Department of Comparative Biomedicine and Food Science (BCA), University of Padua, Viale dell’Università 16, 35020 Legnaro, Italycinzia.centelleghe@unipd.it (C.C.)

**Keywords:** allochthonous, *Trachemys scripta*, testadenovirus, siadenovirus

## Abstract

The spread of non-native freshwater turtles in urban, peri-urban, and natural environments poses increasing ecological and sanitary concerns, particularly due to their potential role as reservoirs of infectious agents. Among these, DNA viruses remain largely unexplored in both invasive and native chelonians. In this study, a molecular survey targeting selected viral pathogens was conducted on oral and cloacal swabs collected from non-native freshwater turtles from natural and confinement ponds in Northeastern Italy, with the aim of assessing the pathogen’s presence and their potential epidemiological relevance. One hundred sixty-four pond sliders (*Trachemys scripta*) were sampled from three sites: Herpesviruses and ranaviruses were not detected; in contrast, adenoviruses were frequently identified (72/163, 44.2%). Sequence analyses allowed their classification mostly as *Testadenovirus trachemys*, with only a single detection of a strain closely related to siadenoviruses and previously associated with mortality events in other tortoise species. Although the pathogenic significance of these viruses remains unclear, their detection highlights the potential role of non-native turtles as viral carriers and underlines the need for systematic virological surveillance in non-native species, particularly in ecosystems shared with susceptible native fauna.

## 1. Introduction

*Trachemys scripta* is a species of pond sliders whose original habitat spans over the Americas; it encompasses three subspecies, namely *T. scripta scripta* (yellow-bellied slider), *T. scripta elegans* (red-eared slider), and *T. scripta troostii* (Cumberland slider) [1]. The rapid diffusion of *T. scripta elegans* outside its natural environment has occurred since the 1960s and peaked in the 90s, when trade was driven by its popularity as a pet, especially in Europe [2]. After an import ban from the European Union (Council Regulation EC No. 388/1997) [3], *T. scripta elegans* was replaced by other subspecies until a definitive ban of the whole species in 2003 (Commission Regulation EC 349/2003) [4]. Concerns about the environmental impact of the introduction of these animals into the wild have risen quickly in different regions due to their great ability to adapt and reproduce in new environments. Notwithstanding these attempts, escaped and illegally released animals have become invasive by reproducing and spreading in the natural environment over the years [5]. The abundant and common presence of *T. scripta* subspecies is attested across Europe in natural and semi-natural contexts [6,7,8,9,10,11], and it is more densely present in northern and central regions of Italy [12,13,14,15,16,17,18,19]. Studies on the impact of this invasive species in new environments have focused mostly on the direct competition with native emydid species (i.e., *Emys orbicularis*) and on its role as a reservoir of zoonotic pathogens [20]. Conversely, its role as a pathogen carrier for other animal species has been less studied by scientific research.

Viruses can play different roles in biological invasions, either decreasing or increasing the impact of their host species. Many viruses infecting hosts in their native range can be transferred along with their introduction into new environments, where they may have a more severe impact on native species if susceptible [21]. Thus, monitoring the health status of invasive species represents a fundamental component of conservation strategies aimed at protecting native species and ecosystems, with a particular attention to pathogens capable of infecting multiple hosts and acting as door openers to secondary infections.

Among the various pathogens, herpesviruses have been frequently detected in chelonians; only two species have been officially recognized so far, infecting preferentially turtles and tortoises, and they have been recently renamed *Scutavirus chelonidalpha5* and *Scutavirus testudinidalpha3* (*Scutavirus* genus, *Alphaherpesvirinae* subfamily, *Orthoherpesviridae* family; https://ictv.global/taxonomy, accessed on 1 March 2026) [22]. Regarding the disease caused by *Scutavirus testudinidalpha3* in Testudinidae, the upper respiratory tract is mainly involved, with diphtheroid necrotizing inflammation as the most characteristic lesion. Vertical transmission has also been proven for this virus [23]. Nonetheless, numerous strains, for which their characterization is still pending, have been identified in various species, witnessing the important viral circulation and genetic variability [22]. Emydidae host a variety of still unclassified strains, which have been identified in asymptomatic animals [24,25] and in the presence of neoplastic [26] or necrotizing [27] lesions, and with other coinfections [28]. Despite similar seasonal fluctuations, a lower infection prevalence was demonstrated in Europe in Emydidae [29,30] compared to the US, with limited detections in *E. orbicularis* and *T. scripta* when compared also to *Terrapene* sp. [29].

Ranaviruses (*Ranavirus* genus, *Alphairidovirinae* subfamily, *Iridoviridae* family; https://ictv.global/taxonomy) are characterized by an important heterogeneity and can be transmitted among different host classes of ectothermic vertebrates [31], complicating their epidemiology. They have been previously associated with mortality events in free-ranging and captive chelonians [32,33,34]. Lethargy, anorexia, respiratory signs, and distress are generally noted [35], and the lesions described in chelonians are periocular, head and extremity swelling, oral cavity ulceration and necrosis, occasional skin ulcerations, and ocular and nasal discharge [33,36]. Subclinical infections have been reproduced experimentally in *T. scripta elegans*, suggesting a role of this species in pathogen dissemination [31]. Notably, the infection severity appears to be modulated by environmental conditions, with lower temperature associated with higher mortality [37]. Ranavirus coinfection with other pathogens is common, complicating the depiction of their pathogenic potential [38].

Another significant viral family affecting reptiles is *Adenoviridae*, which comprises six genera (*Mastadenovirus*, *Aviadenovirus*, *Ichtadenovirus*, *Testadenovirus*, *Barthadenovirus*, and *Siadenovirus*; https://ictv.global/taxonomy), for which evolution has likely occurred in close association with that of their hosts [39], with sporadic host switches. The last three genera have been found in chelonians: Barthadenoviruses have been sporadically detected in tortoises [40,41], testadenoviruses seem to circulate widely in tortoises and turtles, and no clear association with disease has been identified yet [39], whereas siadenoviruses have been associated with mortality events [42]. In this case, the infected tortoises (*Indotestudo forsteni*) presented anorexia, lethargy, oral erosions, nasal and ocular discharge, and diarrhea, with necrotic lesions on multiple internal organs at necropsy [42].

Epidemiological data on these pathogens and hosts are sparse, and the frequency of viral detections can vary among different studies. Surveys have been conducted in Europe, focusing also on autochthonous and endangered species such as the European pond turtle (*E. orbicularis*), and herpesviruses have been detected only sporadically in recent years [29,43]. Ranaviruses seem even less common in freshwater turtles [30,43,44], whereas the presence of adenovirus has been reported in other turtles and tortoises from different European countries [41,44].

Little information is available regarding viral pathogens in the Italian context. Evidence—although scattered—from studies conducted elsewhere has reported their occurrence across multiple host species and different taxonomic classes, as well as the role of viral infections in predisposing hosts to secondary infections. These considerations have prompted the present survey in Northeastern Italy, focusing on allochthonous species that may come into close contact with indigenous ones, thereby representing a potential source of infection and a threat to native ecosystems.

## 2. Materials and Methods

Sites for the epidemiological survey were selected based on the presence of the allochthonous species *T. scripta*. Sites A (Modena, Emilia-Romagna region) and Site B (Rovigo, Veneto region) are regional centers authorized for the confinement of non-native pond sliders: animals originating from the natural or semi-natural environment (Site A) or from private owners (Sites A and B) are brought to the centers and maintained in escape-proof pools lifelong. Site C (Gorizia, Friuli-Venezia Giulia region) represents a protected natural area at the Isonzo river mouth, where an eradication campaign towards exotic emydids is ongoing. In fact, the area represents a Natura 2000 Network site, it is included in the Wetlands of International Importance under the Ramsar Convention (16A02517), and *T. scripta* co-exists in the territory with the native *E. orbicularis* species. Euthanasia of exotic turtles in Site C is therefore authorized and regulated by Istituto Superiore per la Protezione e la Ricerca Ambientale (I.S.P.R.A.) (Rif. int. 30000/2024) in compliance with the Italian law (D. lgs. 230/17).

Sites A and B were selected with the aim of maximizing geographical representativeness, as they included individuals collected across wide areas of Emilia-Romagna and Veneto regions. In contrast, Site C was selected because of its representativeness of a natural environment in which allochthonous species have become established and live in free-ranging conditions.

The minimum sample size was adjusted according to the specific setting. In particular, Sites A and B combined were considered representative of the large population of Emilia-Romagna and Veneto; thus, the required sample size for detecting the presence of a viral agent (i.e., to demonstrate freedom from disease) was 156 individuals, assuming a minimum expected prevalence of 5%, a test sensitivity and specificity of 95% and 98%, respectively, and accepting a type I error of 5% and a type II error of 10%.

Conversely, the estimated closed population size of Site C was approximately 300 individuals. Based on the same assumptions but with a higher expected prevalence of 10%—given the longer residence time in the area and increased contact rates—the minimum required sample size was 52 individuals.

Procedures on live animals were authorized by the university’s ethical committee (code 70/2025). Animals were pulled from the ponds with a fishing net, and oral and cloacal swabs were collected while they were gently restrained by hand. Animals were quickly released to minimize stress after identification with a non-toxic marker to avoid resampling.

Oral and cloacal swabs were collected independently from each animal and processed for separate extractions. Swabs were conferred to the Infectious Disease Laboratory of the Animal Medicine Production and Health (MAPS) Department of Padua University (Legnaro, Italy).

Swabs were individually eluted in 1 mL of 1X PBS and vortexed for 20 s; then, aliquots of 100 µL per sample were pooled by grouping 5 individuals by the matrix, site, and date of sampling in order to maximize the number of tested individuals while still guaranteeing adequate sensitivity.

An aliquot of 200 µL from each pool was processed for DNA extraction using a QIAamp 96 DNA QIAcube HT Kit (Qiagen, Hilden, Germany) on a QIAcube HT automated instrument (Qiagen, Hilden, Germany), following the manufacturer’s instructions. Original and extracted samples were stored at −20 °C until and after processing.

Extracted DNA samples were screened for three viral pathogens, herpesviruses, ranaviruses, and adenoviruses using previously validated molecular methods with slight modifications, adapting protocols to the Platinum™ II Taq Hot-Start DNA Polymerase kit (Invitrogen™, Thermofisher Scientific, Waltham, MA, USA) and SimpliAmp Thermal Cycler instrument (Invitrogen™, Thermofisher Scientific, Waltham, MA, USA).

To detect herpesviruses, a pan-herpesvirus nested PCR targeting the DNA polymerase gene [45] was performed using a feline vaccine as a positive control; to detect ranaviruses, a PCR targeting the MCP gene [46] was performed using a Rana esculenta virus isolate (REV 282/I02) (courtesy of Dr. Anna Toffan, Fish Virology Laboratory, Istituto Zooprofilattico Sperimentale delle Venezie, IZSVe) as a positive control; to detect adenoviruses, a pan-adenovirus nested PCR targeting the DNA polymerase gene [47] was performed using a field fowl adenovirus strain as a positive control [48]. Details on PCR primers and conditions are reported in Supplementary Table S1.

PCR results were analysed by gel electrophoresis on 2% agarose gel stained with SYBR™ Safe DNA Gel Stain (Invitrogen™, Thermofisher Scientific, Waltham, MA, USA). Original samples from positive pools were retrieved and then processed individually for DNA extraction and tested as described above.

Positive individual samples were Sanger sequenced in both directions using the same amplification primer pair at Macrogen Europe (Milan, Italy).

The obtained chromatograms underwent a quality check using FinchTV software v 1.4.0 (Geospiza Inc., Seattle, WA, USA), and forward and reverse sequences were assembled in consensus sequences using ChromasPro 2.1.8 software (Technelysium Pty Ltd., Helensvale, QLD, Australia).

Specificity of consensus sequences was confirmed by BLAST search (https://blast.ncbi.nlm.nih.gov/Blast.cgi, accessed on 1 March 2026), and reference sequences were downloaded from GenBank to perform phylogenetic analyses. Sequences were aligned to the reference set using the MAFFT online version (https://mafft.cbrc.jp/alignment/server/, accessed on 1 March 2026) [49]. Maximum likelihood phylogenetic tree reconstruction was performed using MEGA 12 software [50], choosing the substitution model with the lowest Bayesian information criterion (BIC), and branch support was determined by performing 1000 bootstrap replicates.

## 3. Results

One hundred sixty-four animals from three different collection sites were sampled (Site A = n. 70 individuals; Site B = n. 74 individuals; Site C = n. 20 individuals). No lesions or clinical signs were evidenced during sampling. All individuals belonged to the species *T. scripta*, except for five *Graptemys* sp. (from Site B) and three *Mauremys sinensis* (two from Site A and one from Site B). When possible, the subspecies was identified, and 25 *T. scripta elegans* (5 from Site A; 20 from Site C), 44 *T. scripta scripta* (all from Site A), 6 *T. scripta troostii* (all from Site A) were recorded.

At least one matrix was sampled from each individual, yielding a total of 163 cloacal swabs and 157 oral swabs, which were grouped in 33 cloacal and 32 oral pools. All pools were negative when screened for herpesvirus and ranavirus. On the other hand, 17 cloacal and 19 oral pools were positive for adenovirus. After individual sample testing, 72 animals were positive for adenoviruses, for which both cloacal and oral swabs were available.

Overall, 55 out of 144 animals from Sites A and B, representative of populations from Emilia-Romagna and Veneto regions, tested positive for adenovirus, corresponding to an infection proportion of 38.19% (95% CI: 30.23–46.65%). Forty out of seventy animals from Site A were positive (57.1% [CI: 44.75–68.91%]); 15 out of 74 animals from Site B were positive (20.3% [CI: 11.81–31.22%]); 17 out of 20 animals from Site C were positive (85% [CI: 62.11–96.79%]) (Table 1).

A total of 90 samples were positive; of those, 34 were cloacal swabs and 56 oral swabs. Cloacal and oral swabs were both positive in 18 animals (nine from Site A; five from Site B; four from Site C) (Supplementary Table S1).

**Figure 1:**
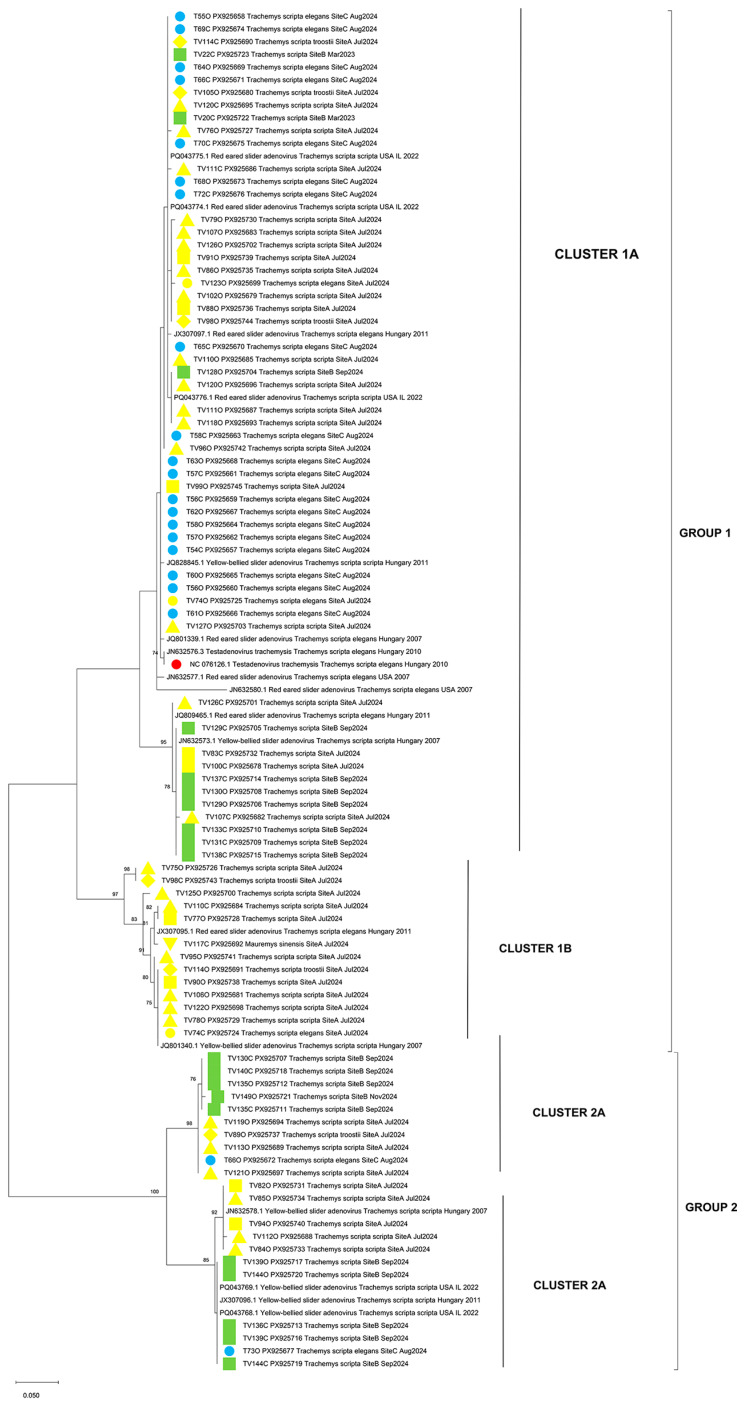
The phylogeny tree for testadenoviruses was reconstructed using the Maximum likelihood method with the Kimura 2-parameter model of nucleotide substitutions and a discrete Gamma distribution. The percentage of replicate trees (>70%) in which the associated taxa clustered together (1.000 bootstrap replicates) is shown above the branches. The complete deletion option was applied to eliminate positions containing gaps and missing data, resulting in a final dataset comprising 251 nucleotide positions. Origin of sequences is color-coded (Site A—yellow; Site B—green; Site C—blue), and host subspecies is shape-coded (downward triangle *M. sinensis*; upward triangle *T. scripta scripta*; circle *T. scripta elegans*; diamond *T. scripta troostii*; square *T. scripta*); reference type species sequence is marked by a red circle.

The amplicon specificity was confirmed by sequencing for all samples. All the identified strains were characterized as *Testadenovirus trachemys* species (Genbank Accession Numbers PX925657–PX925745) (Figure 1; Supplementary Table S2), except for one sequence (Acc. Num. PX963141) from a cloacal swab of a *T. scripta scripta* from Site A, thatshowed 100% identity with Sulawesi tortoise adenovirus 1 (Acc. Num. PQ043778.1) by BLAST search and thus appeared to belong to the genus *Siadenovirus*.

**Figure 2:**
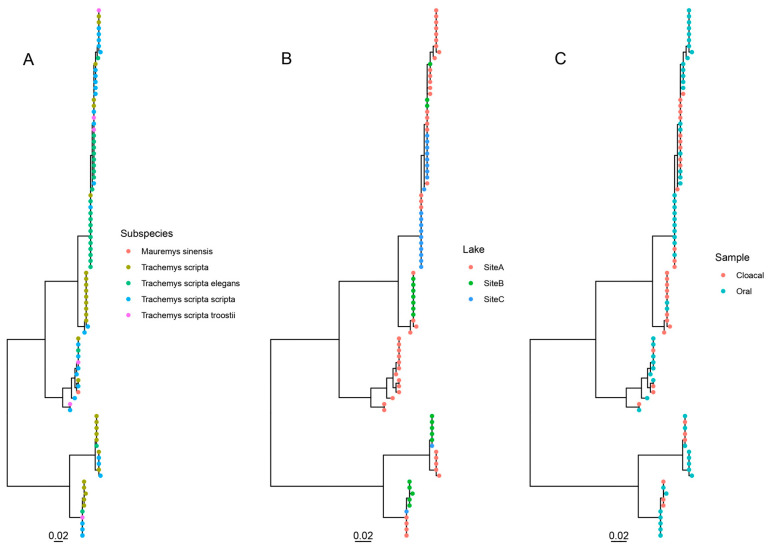
The representation of sequence clustering is highlighted in the different trees by animal subspecies (**A**), site of collection (**B**), and matrix (**C**). Trees were reconstructed using the Maximum likelihood method implemented in MEGA12 [50].

Testadenovirus sequences appeared to split into two major groups, regardless of host subspecies, sampling site, or matrix (Figure 1 and Figure 2).

**Figure 3:**
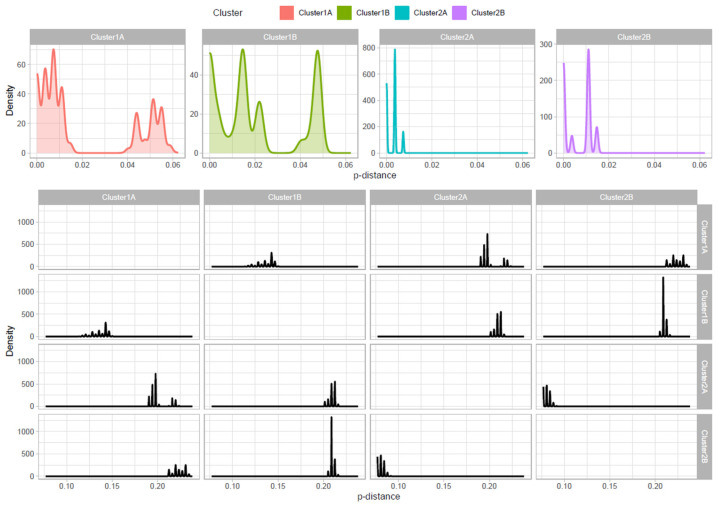
Density plot depicting the pairwise genetic distance (x-axis: p-distance; y-axis: density) calculated within cluster (upper panel) and between clusters (lower panel).

Minor clusters were evidenced within the groups: Group 1 presented a big cluster (Cluster 1A; mean p-distance = 0.0202; min–max = 0–0.0623) comprising two subclusters of sequences sampled across all sites and subspecies and a smaller one (Cluster 1B; mean p-distance = 0.0202; min–max = 0–0.0476) consisting of sequences from Site A only; Group 2 presented two smaller clusters of mixed sequences from all sites and different subspecies (Cluster 2A: mean p-distance = 0.0027; min–max = 0–0.007 and Cluster 2B: mean p-distance = 0.0066; min–max = 0–0.0147) (Figure 3).

Based on location, Site A appeared to host sequences from all clusters; sequences from Site B were present in almost all clusters (except for Cluster 1B), and this pond seemed to host a wide variability of strains, similarly to Site A (Figure 1 and Figure 2). On the other hand, almost all sequences collected from Site C appeared highly similar and belonged to Cluster 1A, except for two sequences in Group 2 (Figure 1 and Figure 2).

Different clusters presented a broad host tropism in terms of subspecies circulation, with Cluster 1B being detected in all subspecies, and Cluster 1A and Group 2 in all subspecies except for *M. sinensis*.

When evaluating variability in relation to subspecies, all species hosted both closely and distantly related strains (reflecting both intra- and inter-cluster variability). Likely because of their common site of origin, *T. scripta elegans* appeared to host the most consistent group of highly similar sequences, whereas the group of sequences with the highest variability were those collected from animals that were not characterized at the subspecies level (Figure 2).

From the 18 animals for which both oral and cloacal swabs were positive, in six cases (35.3%), the obtained sequences were identical (two *T. scripta elegans* from Site C, four *T. scripta* from Site B); in three cases (17.6%), the sequences belonged to the same group and cluster (2 *T. scripta scripta* from Site A, 1 *T. scripta elegans* from Site C); in six cases (35.3%), the sequences belonged to the same group but different clusters (two *T. scripta scripta*, two *T. scripta elegans*, two *T. scripta troostii* all from Site A); in two cases (11.8%), the sequences belonged to two different groups (one *T. scripta* from Site B, one *T. scripta elegans* from Site C). Although a certain degree of clustering could be observed, most sequences originating from the two districts were interspersed within the phylogenetic tree. Remarkably, in a *T. scripta* from Site A (1/18 animals with both swabs positive; 5.9%), a *Testadenovirus trachemys* sequence was identified from the oral swab, and a sequence belonging to genus *Siadenovirus* was identified from the cloacal swab.

## 4. Discussion

The results of the present study did not provide evidence of herpesvirus or ranavirus circulation at Sites A, B, and C. Regarding Sites A and B, although the calculated sample size required to demonstrate freedom from disease at the design prevalence was 156 animals, the observed dataset consisted of 144 tested subjects, all of which yielded negative results. Given the complete absence of positive reactors, the probability of obtaining such a result if the disease were truly present at the design prevalence (5%) was <0.0001. Therefore, despite the slightly smaller sample size than that estimated a priori, the observed data provided sufficient evidence to reject the hypothesis of disease presence at the target prevalence level.

The lack of detections of herpesvirus and ranavirus could be explained by low prevalence in the population and by the latency strategy of herpesvirus, which would have been hardly identifiable without a dedicated sampling of tissues where latency has been proven. Clearly, the random selection of individuals and the lack of evident clinical signs or oral lesions possibly indicating acute or ongoing infections have lowered the likelihood of viral shedding detection. Serological surveys would have been more informative, allowing the evaluation of a previous exposure. However, the availability of diagnostic tools for the indirect detection of these pathogens in such host species remains limited.

Likewise, a study comparing tail-clipping and swabbing suggested the lack of sensitivity of oral and cloacal sampling to detect ranaviruses in turtles [51]. Although different sampling approaches on live animals could have been indeed more informative, especially in cases of subclinical infections [52], they would have been more invasive and hardly practicable from an ethical standpoint. On the other hand, many studies have reported no ranavirus detections in chelonians, also in Europe [43,44,53,54], particularly, in *T. scripta* [30,55], supporting a limited circulation.

While a low infection prevalence could be inferred for herpesviruses and ranaviruses, adenoviruses showed a markedly higher frequency, revealing a different scenario, as previously reported in other areas [39,41,44,56].

The selected pan-adenovirus PCR assay is broadly inclusive, granting a wider screening of an under-investigated viral population, and the possible detrimental effect of primer degeneration on sensitivity is well balanced by reamplification during the nested step. However, PCR is usually regarded as less sensitive than qPCR, and targeting a highly conserved genetic region could have potentially reduced the informativeness of downstream sequencing and genetic analysis.

On the other hand, the identification of two distinct viral genera would not have been possible without such an inclusive approach. A particularly significant finding is represented by the detection of a siadenovirus closely related to viruses previously associated with mass mortality events in Sulawesi tortoises (*Indotestudo forsteni*) [42] and more isolated but related cases in impressed tortoises (*Manouria impressa*) and a Burmese star tortoise (*Geochelone platynota*) [57]. Although no overt clinical signs were evidenced in the sampled individual, nor was mortality previously reported by the pond keepers, the presence of this strain—even at low frequency—should be investigated as a possible risk for the autochthonous species of freshwater turtles and terrestrial tortoises. Given the poorly characterized host range and pathogenic potential of this virus, *Emys* spp. and *Testudo* spp. susceptibility cannot be ruled out.

As reported for testadenoviruses [39], sliders appear less susceptible to disease caused by adenoviruses and may act as reservoirs, as supported by the moderate prevalence previously reported in red-eared sliders in the US [56,58]. Nonetheless, their role in adenovirus introduction in the environment or maintenance could constitute a serious threat to more susceptible species, especially in areas where contact with other emydid or testudinid species is close. In the confinement centers, the escape-proof barriers bordering the pools avoid direct contact with other free-ranging animals in the close vicinity, but viral circulation in natural environments among exotic emydid turtles remains to be further investigated. As a matter of fact, this appears to be the first detection of this virus in Italy, and dedicated studies should be addressed to investigate its geographic and host distribution.

Despite the initial concerns related to the genetic informativeness of the sequenced region, the variability observed among the Testadenovirus strains was nevertheless remarkable and implied a likely wider heterogeneity on a larger genomic scale, that could allow further considerations in terms of classification. No viral clustering emerged in relation to host subspecies, even though the lack of subspecies classification for most of the animals prevents additional evaluations. Furthermore, the detection of a positive individual belonging to *M. sinensis* might suggest a tropism not only limited to the *Trachemys* species but also including at least other members of the Emydidae family. Therefore, host plasticity or broad host tropism can be speculated and should be further explored, particularly in a scenario where *T. scripta* lives in sympatry with the native and locally threatened *E. orbicularis*.

On the other hand, any statement in this sense must be evaluated with caution since molecular detections do not necessarily imply active infection and might reflect contamination among individuals sharing an environment featured by high infectious pressure and animal densities. This could also explain the notable number of animals hosting different strains in the oral cavity and cloaca. In fact, while a third of the cases presented the same strain in oral and cloacal swabs, possibly reflecting true shedding in both matrices and active infection, the presence of different strains in the other animals may instead indicate environmental contamination, consistent with the viral abundance and high genetic heterogeneity present in the ponds. However, frequent co-infection events determined by high infectious pressure cannot be ruled out. The evidence of some district-specific clusters might also allow the speculation of a preferential tropism of some variants, although the study design, the partial genetic information, and the limited sample size warrant caution.

Finally, the simultaneous presence of different strains in a single sample is not usually detectable with common sequencing approaches like those adopted in the present study, except for circumstances where the strains are present in comparable amounts in the same sample. The presence of the same strain in multiple body districts may have been masked by co-infections with other members of the same viral species, thereby increasing the apparent discrepancy in body district–strain/cluster associations.

The remarkable genetic heterogeneity of viral sequences observed in Sites A and B may be attributable to the continuous introduction of new hosts that, for management and environmental protection purposes, are collected from multiple sources and geographic locations, thereby promoting strain mixing. At the same time, the introduction of naive previously captive individuals in confined and densely populated ponds, where high infectious pressure is present, may explain the ongoing viral circulation and evolution.

Despite the limited sample size failing to meet the target required for statistically robust calculations, a different evaluation could be inferred for the Site C population, which can be considered a natural one, since no controlled introductions are made. In fact, the strain variability appeared much lower, likely reflecting viral maintenance in a more stable host population. Conversely, the high rate of positivity in the natural environment, where other emydid species co-exist, is unsettling and should deserve dedicated investigations, especially if similar dynamics can be inferred also for other, more aggressive pathogens. In fact, as an indirect implication of this finding, the intense adenovirus circulation at Site C suggests frequent and effective contacts among individuals, for example, during basking activity, which would also favor the spread of other viral infections reaching moderate to high prevalence. This observation supports the interpretation of a true negative status for the other investigated viruses, even though the target sample size was not fully achieved.

The abundant presence of these viruses in the ponds in all subspecies and the wide heterogeneity herein detected in an otherwise highly conserved genomic region could set the premises for high infectious pressure, rapid evolution, and host adaptation with possible host jumps.

## 5. Conclusions

While testadenoviruses appear common in sliders, their pathogenic role has not been well characterized yet, and data on their virulence in the European pond slider are still lacking; therefore, severe consequences from the infection cannot be ruled out. Investigations on native species, sharing the environment with positive exotic turtles, are therefore advocated. Siadenovirus detection should also be kept into consideration for planning further studies, still based on inclusive assays capable of detecting the circulation of highly divergent viruses. Moreover, the present study investigated only three DNA viruses, leaving a wide array of other agents (including RNA viruses and emerging pathogens) yet to be explored both in terms of prevalence and pathogenic potential; thus, more inclusive and targeted studies on these species are warranted. Such integrated surveillance would not only contribute to early detection and management of biological encroachment, but it could also help mitigate the indirect impacts of pathogen transmission by invasive species to vulnerable taxa. In this context, preserving habitat integrity and maintaining the ecological balance of freshwater environments are essential to safeguard both protected and non-protected native species, which are currently threatened by the combined pressures of invasive competitors and emerging diseases.

## Figures and Tables

**Table 1:** Summary of positive animals based on collection site and taxon (*T.s.* = *Trachemys scripta*; *T. scripta* subsp = animals for which the subspecies was not defined). ^1^ Clopper–Pearson exact method.

Site	Taxon	Positive Animals	Frequency	Prevalence [CI] ^1^
A	*T.s. elegans*	2	2/5	40.0% [5.27–85.34]
	*T.s. scripta*	24	24/44	54.5% [38.77–69.64]
	*T.s. troostii*	4	4/6	66.7% [22.28–95.67]
	*M. sinensis*	1	1/2	50.0% [1.26–98.74]
	*T. scripta* subsp.	9	9/13	69.2% [38.57–90.91]
	Total	40	40/70	57.1% [44.75–68.91]
B	*T. scripta* subsp.	15		
	Total	15	15/74	20.3% [11.81–31.22]
C	*T.s. elegans*	17		
	Total	17	17/20	85% [62.11–96.79]

## Data Availability

Genetic data were uploaded to Genbank (Accession numbers PX925657–PX925745; PX963141).

## Supplementary Materials

The following supporting information can be downloaded at https://www.mdpi.com/article/10.3390/v18060676/s1. Table S1: PCR primers, assay conditions, and references; Table S2: Information summary of animals positive for *Testadenovirus trachemys* with accession numbers (Acc. Num.) of deposited sequences and reference strains.
