# Supplementary material for: Epidemiological Survey of DNA Viruses in Non-Native Pond Sliders (Trachemys scripta) in Northeastern Italy
Source: Viruses. 2026 Jun 17;18(6):676. doi: 10.3390/v18060676 (PMC13307794; doi:10.3390/v18060676)
Supplement: Supplementary file 1 [file viruses-18-00676-s001.zip › Supplementary table S1.pdf]

Supplementary Table S1: PCR primers, assay conditions and references.

**Herpesvirus assay**			
**PCR1 Primers**	**Thermal protocol**		
**DFA 5′-GAYTTYGCNAGYYTNTAYCC-3′**	94°C	2min	
**ILK 5′-TCCTGGACAAGCAGCARNYSGCNMTNAA-3′**	94°C	15sec	
**KG1 5′-GTCTTGCTCACCAGNTCNACNCCYTT-3′**	46°C	15sec	45
	68°C	10sec	
	68°C	1min	
**PCR2 Primers**	**Thermal protocol**		
**TGV 5′-TGTAACTCGGTGTAYGGNTTYACNGGNGT-3′**	94°C	2min	
**IYG 5′-CACAGAGTCCGTRTCNCCRTADAT-3′**	94°C	15sec	
	46°C	15sec	45
	68°C	10sec	
	68°C	1min	
VanDevanter, D. R., Warrener, P., Bennett, L., Schultz, E. R., Coulter, S., Garber, R. L., & Rose, T. M. (1996). Detection and analysis of diverse herpesviral species by consensus primer PCR. Journal of clinical microbiology, 34(7), 1666-1671.			
**Ranavirus assay**			
**PCR Primers**	**Thermal protocol**		
**RanaMCPstdF 5′-GTTCTCACACGCAGTCAAGG-3′**	94°C	2min	
**RanaMCPstdR 5′-CGGACAGGGTGACGTTAAG-3′**	94°C	15sec	
	50°C	15sec	45
	68°C	10sec	
	68°C	1min	
Stilwell, N. K., Whittington, R. J., Hick, P. M., Becker, J. A., Ariel, E., Van Beurden, S., ... & Waltzek, T. B. (2018). Partial validation of a TaqMan real-time quantitative PCR for the detection of ranaviruses. Diseases of aquatic organisms, 128(2), 105-116.			
**Adenovirus assay**			
**PCR1 Primers**	**Thermal protocol**		
**polFouter 5′-TNMGNGGNGGNMGNTGYTAYCC-3′**	94°C	2min	
**polRouter 5′-GTDGCRAANSHNCCRTABARNGMRTT-3′**	94°C	15sec	
	55°C	15sec	45
	68°C	15sec	
	68°C	1min	
**PCR2 Primers**	**Thermal protocol**		
**polFinner 5′-GTNTWYGAYATHTGYGGHATGTAYGC-3′**	94°C	2min	
**polRinner 5′-CCANCCBCDRTTRTGNARNGTRA-3′**	94°C	15sec	
	55°C	15sec	45
	68°C	7sec	
	68°C	1min	
Wellehan, J. F., Johnson, A. J., Harrach, B., Benkö, M., Pessier, A. P., Johnson, C. M., ... & Jacobson, E. R. (2004). Detection and analysis of six lizard adenoviruses by consensus primer PCR provides further evidence of a reptilian origin for the atadenoviruses. Journal of virology, 78(23), 13366-13369.			
