# Supplementary material for: Epidemiological Survey of DNA Viruses in Non-Native Pond Sliders (Trachemys scripta) in Northeastern Italy
Source: Viruses. 2026 Jun 17;18(6):676. doi: 10.3390/v18060676 (PMC13307794; doi:10.3390/v18060676)
Supplement: Supplementary file 1 [file viruses-18-00676-s001.zip › Supplementary table S2.pdf]

Supplementary Table S2: Information summary of animals positive for *testadenovirus trachemys* with accession numbers (Acc. Num.) of deposited sequences and reference strains.

Sequence Id	Acc. Num.	Collection Date	Host	Collection Site	Isolation Source
**T54C**	PX925657	August 2024	*T. scripta elegans*	Site C	cloacal swab
**T55O**	PX925658	August 2024	*T. scripta elegans*	Site C	oral swab
**T56C**	PX925659	August 2024	*T. scripta elegans*	Site C	cloacal swab
**T56O**	PX925660	August 2024	*T. scripta elegans*	Site C	oral swab
**T57C**	PX925661	August 2024	*T. scripta elegans*	Site C	cloacal swab
**T57O**	PX925662	August 2024	*T. scripta elegans*	Site C	oral swab
**T58C**	PX925663	August 2024	*T. scripta elegans*	Site C	cloacal swab
**T58O**	PX925664	August 2024	*T. scripta elegans*	Site C	oral swab
**T60O**	PX925665	August 2024	*T. scripta elegans*	Site C	oral swab
**T61O**	PX925666	August 2024	*T. scripta elegans*	Site C	oral swab
**T62O**	PX925667	August 2024	*T. scripta elegans*	Site C	oral swab
**T63O**	PX925668	August 2024	*T. scripta elegans*	Site C	oral swab
**T64O**	PX925669	August 2024	*T. scripta elegans*	Site C	oral swab
**T65C**	PX925670	August 2024	*T. scripta elegans*	Site C	cloacal swab
**T66C**	PX925671	August 2024	*T. scripta elegans*	Site C	cloacal swab
**T66O**	PX925672	August 2024	*T. scripta elegans*	Site C	oral swab
**T68O**	PX925673	August 2024	*T. scripta elegans*	Site C	oral swab
**T69C**	PX925674	August 2024	*T. scripta elegans*	Site C	cloacal swab
**T70C**	PX925675	August 2024	*T. scripta elegans*	Site C	cloacal swab
**T72C**	PX925676	August 2024	*T. scripta elegans*	Site C	cloacal swab
**T73O**	PX925677	August 2024	*T. scripta elegans*	Site C	oral swab
**TV100C**	PX925678	July 2024	*T. scripta*	Site A	cloacal swab
**TV102O**	PX925679	July 2024	*T. scripta scripta*	Site A	oral swab
**TV105O**	PX925680	July 2024	*T. scripta troostii*	Site A	oral swab
**TV106O**	PX925681	July 2024	*T. scripta scripta*	Site A	oral swab
**TV107C**	PX925682	July 2024	*T. scripta scripta*	Site A	cloacal swab
**TV107O**	PX925683	July 2024	*T. scripta scripta*	Site A	oral swab
**TV110C**	PX925684	July 2024	*T. scripta scripta*	Site A	cloacal swab
**TV110O**	PX925685	July 2024	*T. scripta scripta*	Site A	oral swab
**TV111C**	PX925686	July 2024	*T. scripta scripta*	Site A	cloacal swab
**TV111O**	PX925687	July 2024	*T. scripta scripta*	Site A	oral swab
**TV112O**	PX925688	July 2024	*T. scripta scripta*	Site A	oral swab
**TV113O**	PX925689	July 2024	*T. scripta scripta*	Site A	oral swab
**TV114C**	PX925690	July 2024	*T. scripta troostii*	Site A	cloacal swab
**TV114O**	PX925691	July 2024	*T. scripta troostii*	Site A	oral swab
**TV117C**	PX925692	July 2024	*Mauremys sinensis*	Site A	cloacal swab
**TV118O**	PX925693	July 2024	*T. scripta scripta*	Site A	oral swab
**TV119O**	PX925694	July 2024	*T. scripta scripta*	Site A	oral swab
**TV120C**	PX925695	July 2024	*T. scripta scripta*	Site A	cloacal swab
**TV120O**	PX925696	July 2024	*T. scripta scripta*	Site A	oral swab
**TV121O**	PX925697	July 2024	*T. scripta scripta*	Site A	oral swab
**TV122O**	PX925698	July 2024	*T. scripta scripta*	Site A	oral swab
**TV123O**	PX925699	July 2024	*T. scripta elegans*	Site A	oral swab
**TV125O**	PX925700	July 2024	*T. scripta scripta*	Site A	oral swab
**TV126C**	PX925701	July 2024	*T. scripta scripta*	Site A	cloacal swab
**TV126O**	PX925702	July 2024	*T. scripta scripta*	Site A	oral swab

**TV127O**	PX925703	July 2024	*T. scripta scripta*	Site A	oral swab
**TV128O**	PX925704	September 2024	*T. scripta*	Site B	oral swab
**TV129C**	PX925705	September 2024	*T. scripta*	Site B	cloacal swab
**TV129O**	PX925706	September 2024	*T. scripta*	Site B	oral swab
**TV130C**	PX925707	September 2024	*T. scripta*	Site B	cloacal swab
**TV130O**	PX925708	September 2024	*T. scripta*	Site B	oral swab
**TV131C**	PX925709	September 2024	*T. scripta*	Site B	cloacal swab
**TV133C**	PX925710	September 2024	*T. scripta*	Site B	cloacal swab
**TV135C**	PX925711	September 2024	*T. scripta*	Site B	cloacal swab
**TV135O**	PX925712	September 2024	*T. scripta*	Site B	oral swab
**TV136C**	PX925713	September 2024	*T. scripta*	Site B	cloacal swab
**TV137C**	PX925714	September 2024	*T. scripta*	Site B	cloacal swab
**TV138C**	PX925715	September 2024	*T. scripta*	Site B	cloacal swab
**TV139C**	PX925716	September 2024	*T. scripta*	Site B	cloacal swab
**TV139O**	PX925717	September 2024	*T. scripta*	Site B	oral swab
**TV140C**	PX925718	September 2024	*T. scripta*	Site B	cloacal swab
**TV144C**	PX925719	September 2024	*T. scripta*	Site B	cloacal swab
**TV144O**	PX925720	September 2024	*T. scripta*	Site B	oral swab
**TV149O**	PX925721	November 2024	*T. scripta*	Site B	oral swab
**TV20C**	PX925722	March 2023	*T. scripta*	Site B	cloacal swab
**TV22C**	PX925723	March 2023	*T. scripta*	Site B	cloacal swab
**TV74C**	PX925724	July 2024	*T. scripta elegans*	Site A	cloacal swab
**TV74O**	PX925725	July 2024	*T. scripta elegans*	Site A	oral swab
**TV75O**	PX925726	July 2024	*T. scripta scripta*	Site A	oral swab
**TV76O**	PX925727	July 2024	*T. scripta scripta*	Site A	oral swab
**TV77O**	PX925728	July 2024	*T. scripta*	Site A	oral swab
**TV78O**	PX925729	July 2024	*T. scripta scripta*	Site A	oral swab
**TV79O**	PX925730	July 2024	*T. scripta scripta*	Site A	oral swab
**TV82O**	PX925731	July 2024	*T. scripta*	Site A	oral swab
**TV83C**	PX925732	July 2024	*T. scripta*	Site A	cloacal swab
**TV84O**	PX925733	July 2024	*T. scripta scripta*	Site A	oral swab
**TV85O**	PX925734	July 2024	*T. scripta scripta*	Site A	oral swab
**TV86O**	PX925735	July 2024	*T. scripta scripta*	Site A	oral swab
**TV88O**	PX925736	July 2024	*T. scripta*	Site A	oral swab
**TV89O**	PX925737	July 2024	*T. scripta troostii*	Site A	oral swab
**TV90O**	PX925738	July 2024	*T. scripta*	Site A	oral swab
**TV91O**	PX925739	July 2024	*T. scripta*	Site A	oral swab
**TV94O**	PX925740	July 2024	*T. scripta*	Site A	oral swab
**TV95O**	PX925741	July 2024	*T. scripta scripta*	Site A	oral swab
**TV96O**	PX925742	July 2024	*T. scripta scripta*	Site A	oral swab
**TV98C**	PX925743	July 2024	*T. scripta troostii*	Site A	cloacal swab
**TV98O**	PX925744	July 2024	*T. scripta troostii*	Site A	oral swab
**TV99O**	PX925745	July 2024	*T. scripta*	Site A	oral swab
**REFERENCES**					
	JN632573.1	2007	*T. scripta scripta*	Hungary	
	JN632576.3	2010	*T. scripta elegans*	Hungary	
	JN632577.1	2007	*T. scripta elegans*	USA	
	JN632578.1	2007	*T. scripta scripta*	Hungary	
	JN632580.1	2007	*T. scripta elegans*	USA	

	JQ801339.1	2007	*T. scripta elegans*	Hungary
	JQ801340.1	2007	*T. scripta scripta*	Hungary
	JQ809465.1	2011	*T. scripta elegans*	Hungary
	JQ828845.1	2011	*T. scripta scripta*	Hungary
	JX307095.1	2011	*T. scripta elegans*	Hungary
	JX307096.1	2011	*T. scripta scripta*	Hungary
	JX307097.1	2011	*T. scripta elegans*	Hungary
	NC 076126.1	2010	*T. scripta elegans*	Hungary
	PQ043768.1	2022	*T. scripta scripta*	USA
	PQ043769.1	2022	*T. scripta scripta*	USA
	PQ043774.1	2022	*T. scripta scripta*	USA
	PQ043775.1	2022	*T. scripta scripta*	USA
	PQ043776.1	2022	*T. scripta scripta*	USA
